# Concise Review: Deciphering the Mechanism Behind Induced Pluripotent Stem Cell Generation

**DOI:** 10.1002/stem.744

**Published:** 2011-09-21

**Authors:** Shi-Lung Lin

**Affiliations:** Division of Regenerative Medicine, WJWU & LYNN Institute for Stem Cell ResearchSanta Fe Springs, California, USA

**Keywords:** Induced pluripotent stem cell, Mechanism, Somatic cell reprogramming, DNA demethylation, Pluripotency, miR-302, microRNA

## Abstract

Regenerative medicine using spluripotent/multipotent stem cells holds a great promise in developing therapies for treating developmental abnormalities, degenerative disorders, and aging-related illness. However, supply and safety of the stem cells are two major problems with today's regenerative medicine. Recent development of induced pluripotent stem cells (iPSCs) has overcome the supply shortages by allowing the reprogramming of patients' body cells to embryonic stem cell (ESC)-like pluripotent cells. Still, the potential tumorigenicity of iPSCs remains as an obstacle. During early embryogenesis ESCs can be generated without tumor formation; therefore, understanding the mechanisms underlying ESC generation may help us to prevent iPSC tumorigenicity. Previous studies have shown that an ESC-enriched noncoding RNA, miR-302, induces somatic cell reprogramming (SCR) to form iPSCs, suggesting its pivotal role in stem cell generation. Recent research further revealed that miR-302-induced SCR involves an epigenetic reprogramming mechanism similar to the natural zygotic reprogramming process in the two- to eight-cell-stage embryos. These findings indicate that miR-302, as a cytoplasmic gene silencer, inhibits the translation of multiple key epigenetic regulators, including AOF1/2, methyl-CpG binding proteins 1 and 2, and DNA (cytosine-5-)-methyltransferase 1, to induce global DNA demethylation, which subsequently triggers the activation of the previously defined factors Oct4, Sox2, and Nanog to complete the reprogramming process. The same mechanism was also found in the event of somatic cell nuclear transfer. Based on these advanced understandings, this review describes the currently established SCR mechanism—as compared to the natural process of early ESC formation—and demonstrates how stem cell researchers may use this mechanism to improve iPSC generation.

In the past, it was widely assumed that a stem cell, once differentiated, could not revert back to an earlier developmental stage. The recent discovery of iPSCs, however, revokes this concept and provides the first evidence that there is an internal mechanism capable of reprogramming the “stemness” of a differentiated tissue cell back to an ESC-like pluripotent state, indicating a fountain of youth intrinsic to every cell in the body. A stem cell has two principal abilities of stemness: (1) self-renewal in which it can multiply through unlimited division and (2) pluripotency in which it can differentiate into a variety of tissue cells originating from all three embryonic germ layers, the ectoderm, mesoderm, and endoderm. The breakthrough discovery of such a reprogramming event provides us a powerful means to generate and regenerate unlimited pluripotent stem cells directly from the resourceful pool of body tissue cells. Yet, the involved mechanism, called somatic cell reprogramming (SCR), remains elusive.

SCR was first observed by transferring somatic cell nuclei into the cytoplasm of oocytes, which forms ESC-like hybrid cells that can develop into animal clones possessing the same genetic traits as the hosts of the somatic cell nuclei [1, 2]. Although this kind of somatic cell nuclear transfer (SCNT) technology has been intensively practiced for over 14 years to produce various species of animal clones, the necessity of oocytes is ethically controversial and the mechanism is unclear. In 2006, Takahashi and Yamanaka established a novel reprogramming method that bypassed any use of oocyte or embryonic components. By introducing four defined transcription factors, Oct4, Sox2, Klf4, and c-Myc, somatic cells were reprogrammed to iPSCs that showed ESC-like properties in almost all aspects [3]. Subsequently, Yu et al. [4] also successfully generated iPSCs using another set of four defined factors, Oct4, Sox2, Nanog, and Lin28. Nevertheless, with all these efforts, the SCR mechanism is still unsolved.

It was not until 2 years after the discovery of iPSCs when another method of iPSC generation was found which revealed the mechanism of SCR. Lin et al. and their peers showed that a small noncoding RNA, called miR-302, can replace all previously defined factors to reprogram human and mouse somatic cells to ESC-like iPSCs [5–8]. It was understood that none of these human iPSCs induced by miR-302 have been tested for germline transmission in either chimera or clones because of strong ethical concerns. MiR-302 is a 23-ribonucleotide microRNA (miRNA) expressed abundantly in human ESCs but is absent in all differentiated tissue cells [9]. Despite its presence in ESCs, how does a small RNA, incapable of encoding any protein or peptide, play such a pivotal role in regulating SCR? It turns out that miR-302 functions as a gene silencer and simultaneously downregulates multiple key epigenetic regulators, including lysine-specific histone demethylases 1 and 2 (namely AOF2/1, LSD1/2, or KDM1/1B), DNA (cytosine-5-)-methyltransferase 1 (DNMT1), and methyl-CpG binding proteins 1 and 2 (MECP1/2) [5, 6]. Silencing of these epigenetic regulators induces global DNA demethylation, the first sign of SCR. Like a lock, DNA methylation sets up different somatic gene expression patterns in cells and defines the cells' properties; the key to SCR, global demethylation, unlocks and resets these differentiated gene expression patterns to a highly uniform ESC-like profile. This “unlocking” of a genome allows transcription machinery access to the ESC-specific genes and is required for iPSC formation.

None of the previously defined transcription factors provided an explanation for the process of global DNA demethylation. To clarify this unsolved mechanism, Lin's studies revealed that miR-302 binds to the gene transcripts (mRNAs) of AOF2/1 and MECP1/2 and forms a RNA-induced silencing complex with argonaute proteins and Dicer endoribonucleases to repress the translation of these important epigenetic regulators [5, 6]. In the absence of AOF1 protein synthesis, germ cells do not undergo de novo DNA methylation during oogenesis [10], while AOF2 deficiency causes embryonic lethality due to a progressive loss of genomic DNA methylation and therefore lack of cell differentiation [11]. Hence, silencing of AOF1 and/or 2 is sufficient to induce global demethylation. Silencing of MECP1/2 further enhances this demethylation effect of AOF1/2 deficiency [5, 6].

In addition to global demethylation, Lin's studies also found that miR-302-targeted AOF2 silencing can destabilize DNMT1 activity and prevent replication-dependent DNA methylation during iPSC division, resulting in a passive demethylation mechanism [6]. Based on the analytic results of online miRNA-target prediction program provided by the European Bioinformatics Institute EMBL-EBI (http://www.ebi.ac.uk/enright-srv/microcosm/cgi-bin/targets/v5/detail_view.pl?transcript_id=ENST00000359526), miR-302 also directly targets DNMT1 for gene silencing. Given that DNMT1 functions to maintain inherited CpG methylation patterns by methylating the newly replicated DNA during S phase of the cell cycle, deficiency of its activity has been shown to cause passive DNA demethylation in early zygotic cells during embryonic development [12–15]. Taken together, all of the above studies indicate that SCR involves a passive global demethylation mechanism. However, this passive demethylation model will generate two hemimethylated cells in every single iPSC colony, an event that has not yet been reported. Passive demethylation is unable to remove the methylated sites originally left in the somatic genome before SCR; therefore, only the newly divided daughter cell genomes are demethylated and reprogrammed. Whether these hemimethylated cells are quickly degraded via programmed cell death (apoptosis) during SCR or further demethylated by an active mechanism remains to be determined.

Global demethylation is not a new biological event in view of natural embryonic development. Global demethylation occurs twice during development: first during migration of primordial germ cells (PGCs) into the embryonic gonads (approximately embryonic day E10.5 to E13.5) and next in the one-to-eight-cell-stage zygotic cells after fertilization [13, 14, 16, 17]. Parental methylation imprints are erased and re-established in germline PGCs but largely preserved in postfertilized zygotic cells [18–20], indicating that the germline and zygotic demethylation mechanisms are not identical. Recent discovery of global demethylation in iPSCs reveals a new kind of artificial SCR demethylation mechanism comparable to the natural ones [6]. Similar to zygotic demethylation, SCR demethylation triggers a massive erasure of genomic methylation sites but preserves parental imprints (Fig. 1). However, unlike zygotes, somatic cells do not receive parental elements from oocytes or sperms, such as paternal protamines, maternal proteins, and RNAs. As a result, some zygotic components required for the completion of epigenetic reprogramming during early embryogenesis are missing in SCR. This observation is further supported by recent evidence that the reprogramming induced by SCNT results in more similar epigenomic modification and transcriptomic expression patterns to those of ESCs than does the direct reprogramming by four previously defined factors [21]. Prolonging iPSC culture or repeating the differentiation–reprogramming process may improve the clearance of somatic methylation residuals in iPSCs, yet their pluripotency is still not better than that of SCNT-induced stem cells. Therefore, iPSCs may present certain defects due to their lack of some zygotic components. Given that these zygotic components originate in parental germline cells, recent finding of iPSC-derived PGC-like cells that can be processed through germline demethylation may lead to a solution to this problem [22].

**Figure 1:**
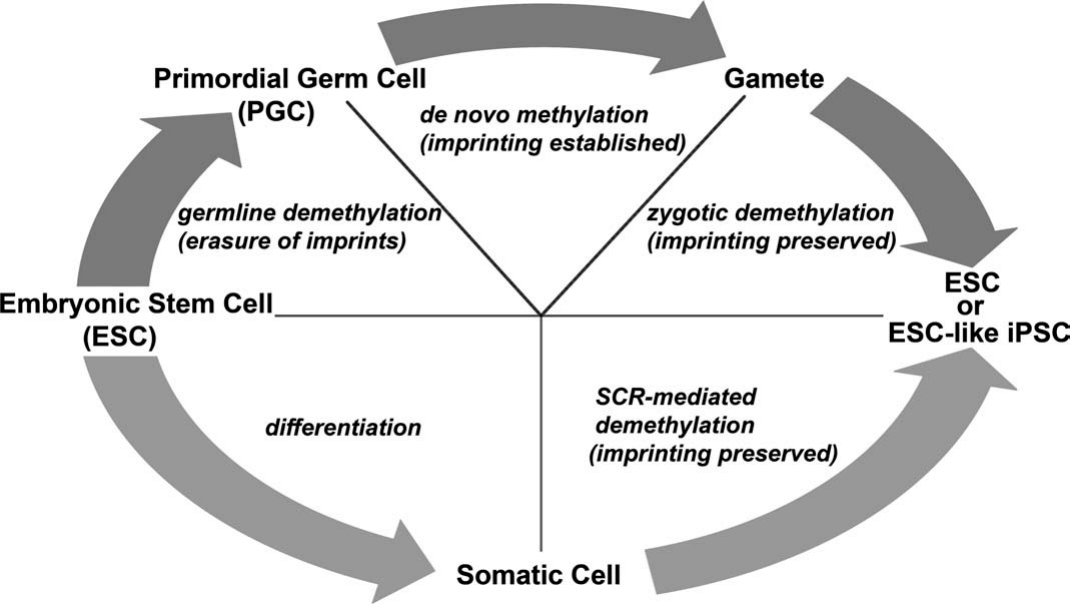
Comparison of natural germline-zygote and manually forced SCR demethylation pathways. Global demethylation occurs naturally in two developmental stages, one during formation and migration of PGCs into the embryonic gonads (germline demethylation) and a second time in one-to-eight-cell-stage zygotes before the morula stage (zygotic demethylation). Parental imprints are erased and re-established during germline demethylation but not in zygotic demethylation, resulting in different genomic imprinting patterns from the parents. On the other hand, SCR demethylation can be forced in somatic cells by manually introducing the expression of either miR-302 or four defined reprogramming factors (Oct4–Sox2–Klf4-c–Myc or Oct4–Sox2–Nanog–Lin28). Notably, Oct4 and Sox2 can also induce miR-302 expression. Because of the effects of miR-302-targeted epigenetic gene silencing, the somatic cell genome is forcedly demethylated without the erasure of parental imprints. As a result, expression of parental germline elements is still inactivated by the imprints. Given that parental elements are essential for normal zygotic development, their deficiency may cause developmental defects in SCR-induced iPSCs. Abbreviations: ESCs, embryonic stem cells; iPSCs, induced pluripotent stem cells; PGCs, primordial germ cell; SCR, somatic cell reprogramming.

After the revelation of miR-302-mediated global demethylation, the next question is how this mechanism relates to the reprogramming induced by previously defined four factors, Oct4, Sox2, Naong, and Lin28. Klf4 and c-Myc are not preferred in the process of iPSC generation due to their potential oncogenic activities. Global demethylation has been reported to promote Oct4–Nanog overexpression in mouse embryos and mouse–human fused heterokaryons [23, 24]. Lin's studies further demonstrated that elevated miR-302 expression to over 1.1–1.3-fold of the normal human ESC level (∼0.9–1.0 million copies per ESC) triggers both global demethylation and coexpression of Oct4, Sox2, and Nanog in human iPSCs [6, 25]. The expression of Lin28 and many other ESC marker genes was observed 1–3 days later than the presence of Oct4–Sox2–Nanog elevation. A similar miR-302 transfection approach was also shown to increase Oct4 and Nanog expression by twofold in human ESCs [26]. Further research in these ESCs revealed that miR-302 directly silences nuclear receptor subfamily 2, group F, number two, a transcriptional repressor against Oct4 expression, to activate Oct4 transcription [27]. These findings have clearly confirmed that global demethylation activates ESC-specific gene expression in particular, Oct4, Sox2, and Nanog. Through silencing of AOF1/2, MECP1/2, and DNMT1, we now understand that miR-302 induces global demethylation and subsequently leads to Oct4–Sox2–Nanog activation essential for the initiation of SCR to form iPSCs. In addition, Oct4 and Sox2 have been found to be the transcriptional activators for miR-302 expression [28–30]. As shown in Figure 2, the mutual stimulation between miR-302 and Oct4–Sox2–Nanog forms a positive feedback regulation loop to maintain the pluripotent status of reprogrammed iPSCs. This finding explains why miR-302, Oct4, Sox2, and Nanog are all essential markers for human ESCs.

**Figure 2:**
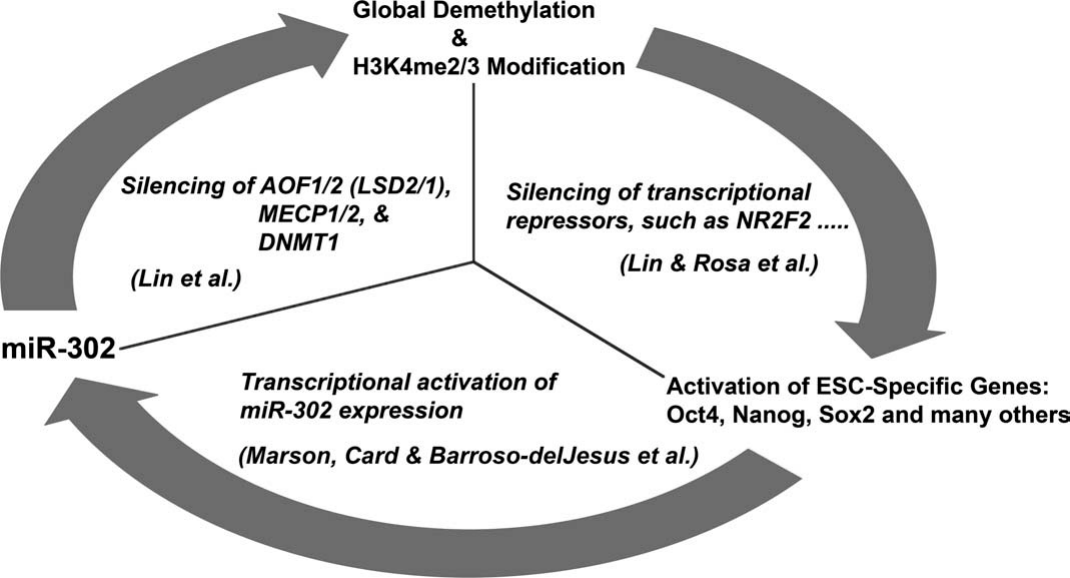
Currently established somatic cell reprogramming (SCR) mechanism for induced pluripotent stem cell (iPSC) generation. MiR-302 silences AOF1/2 and DNMT1 activities and, in conjunction with the cosuppression of MECP1/2 and HDAC2, results in global DNA demethylation and chromosomal H3K4me2/3 modification. Subsequently, these epigenetic reprogramming events induce ESC-specific gene expression, in particular Oct4, Sox2, and Nanog, which in turn further stimulate miR-302 expression to form a positive feedback loop cycle crucial for the maintenance of SCR. Based on this mechanistic model, there are two methods for inducing iPSC formation: one is to force the miR-302 expression and the other is to introduce the coexpression of four defined factors (Oct4–Sox2–Nanog–Lin28). Both methods can trigger the activation of this cycling SCR mechanism; however, miR-302 directly induces global demethylation to initiate SCR while the four factors indirectly function through miR-302 expression. Abbreviations: DNMT1, DNA (cytosine-5-)-methyltransferase one; ESC, embryonic stem cell; MECP1/2, methyl-CpG binding proteins 1 and 2; NR2F2, nuclear receptor subfamily 2, group F, number two.

How can we test the interaction between miR-302 and global demethylation? As global DNA demethylation occurs in the cell nucleus while miR-302 functions in the cytoplasm, SCNT is the best method to demonstrate this epigenetic reprogramming mechanism. SCNT is a well-established technology to generate ESC-like pluripotent stem cells by transferring a somatic cell nucleus into the MII-stage oocyte cytoplasm [1, 2]. Following the same procedure but instead of oocyte cytoplasm, we transferred somatic cell nuclei into the cytoplasm of denucleated miR-302-induced iPSCs. Most (93%) of the hybrid cells were reprogrammed to iPSC-like pluripotent cells possessing ESC-like properties [6]. Global demethylation was also quickly detected a few days after SCNT, indicating that the reprogramming factors in iPSCs are functionally similar to those found in oocyte cytoplasm. Conversely, transferring iPSC nuclei into the somatic cell cytoplasm failed to form any viable cell. Hence, the earliest reprogramming effector, the “initiator,” lies in the cytoplasm rather than nucleus of an iPSC. This finding is coincident with the previous SCNT results using the oocyte cytoplasm, which contains several miR-302 homologs that may have the same reprogramming function, such as miR-200c and miR-371–373. Given that miR-302 is a cytoplasmic effector whereas Oct4, Sox2, and Nanog are all nuclear transcription factors, it is conceivable that miR-302 is responsible for initiating SCR through global demethylation in the SCNT-induced iPSCs. On the other hand, the transfer of iPSC nuclei fails to induce pluripotency, suggesting that somatic cytoplasm may lack a key reprogramming effector required for iPSC generation. Alternatively, somatic cytoplasm may contain an inhibitor directed against iPSC formation; however, this possibility has been ruled out by further induction of miR-302 elevation in the hybrid cells containing the miR-302-induced iPSC nuclei, showing that the efficiency of iPSC generation is proportional to the extent of induced miR-302 elevation [6]. Because of this new SCNT approach, miR-302-induced iPSCs may be applied to replace oocytes to avoid potential ethical concerns.

Regenerative medicine presents the key to future therapies for treating genetic disorders, physical abnormalities, injures, cancers/tumors, and aging. Supply and safety of the stem cells are two of the most important bottlenecks in regenerative medicine. Prior efforts have succeeded in isolating various pluripotent/multipotent stem cells from embryos, umbilical cords/amniotic fluids, and reproductive organs; yet, none of these stem cells can be made in a large quantity enough for patients. Without knowledge of the mechanism underlying stem cell generation, it is difficult to produce sufficient pluripotent stem cells for regenerative medicine. Following the recent identification of miR-302-mediated SCR mechanism (Fig. 2), an induced reprogramming process mimicking the natural zygotic development event, we have now insight into the regulatory mechanism necessary for stem cell generation and a method of reprogramming somatic cells to ESC-like iPSCs. This SCR mechanism is not only capable of generating sufficient iPSCs but also preventing tumor formation in these iPSCs [25]. During SCR, miR-302 functions to silence both cyclin E-CDK2 and cyclin D-CDK4/6 cell cycle pathways during G1–S-phase transition, consequently preventing iPSC tumorigenicity. Furthermore, miR-302 also silences polycomb ring finger oncogene BMI1, a cancer stem cell marker, to promote the expression of two tumor suppressor genes, p16Ink4a and p14/p19Arf. The combination of these two tumor suppression effects results in a tightly regulated cell cycle rate similar to that of the one-to-eight-cell-stage embryonic cells in early mammalian zygotes (20–24 hours per cycle). As human ESCs express abundant miR-302, this tumor suppressor function of miR-302 may explain how early embryonic development circumvents tumor formation. As a result, we may use this new finding for developing tumor-free iPSCs to improve the safety of regenerative medicine.

The mechanism of miR-302-induced SCR presents several advantages over the currently established four-factor induction methods. First, miR-302 directly silences its targeted epigenetic genes and induces global DNA demethylation to initiate epigenetic reprogramming, whereas Oct4, Sox2, and Nanog may indirectly function through miR-302 to continue the reprogramming process. Previous studies have shown that miR-302-induced iPSC generation takes shorter time than does the four-factor induction (∼ 1–2 weeks vs. 2–3 weeks) [5–7]. Second, delivery of a single small miR-302 is significantly easier and more efficient than cotransfecting four large transcription factors. The optimal reprogramming efficiency reported for miR-302 and four-factor induction methods is >10% and <1%, respectively [3, 4, 6, 7]. Third, some of the previously defined four factors are potential oncogenes, such as c-Myc and Klf4. Based on the analytic results of the online miRNA-target prediction program PICTAR-VERT (http://pictar.mdc-berlin.de/), Klf4 is actually a target of miR-367, a miR-302 homolog naturally expressed in the miR-302 familial cluster. Finally, miR-302-induced SCR is compatible with SCNT technologies. Taken together, miR-302 may replace all previously defined reprogramming factors for safer and more efficient iPSC generation.

Five years have elapsed since the initial discovery of iPSC generation to the disclosure of its underlying mechanism, which lead to the understanding of the process of ESC-like pluripotent stem cell generation and regeneration. As previous studies were all performed in isolated cells under in vitro conditions, extending these findings to in vivo applications will be the next challenge. Rejuvenation involves a systemic stem cell regeneration process throughout the whole body. This process requires a large stem cell supply and that must be maintained for a relatively long time. Hence, supply and safety of the used stem cells will significantly affect the final results of rejuvenation. Fortunately, plentiful body cells can now serve as an ideal resource for iPSC generation using miR-302, the introduction of which also prevents tumor formation. In view of these current advances in iPSC generation, we found that natural zygotic reprogramming has offered us a solution to the problems of stem cell supply and safety. This solution relies on a miR-302-induced SCR mechanism similar to zygotic reprogramming during early embryogenesis.

For modern regenerative medicine, the dual function of miR-302 in both reprogramming and tumor suppression has provided us a convenient means to control the quantity and quality of iPSCs, opening up a new avenue toward the fountain of youth. Recent identification of the same reprogramming and tumor suppression function preserved in the cell lysates of these miR-302-induced iPSCs further leads to a novel SCNT application for iPSC generation without using any transgenic or genomic materials. Based on these groundbreaking discoveries, the development of a feasible strategy and therapy for not only local regenerative medicine but also whole systemic rejuvenation is highly expected in the near future.
